# A novel model to study the impact of gender-affirming therapy on bone in young male mice

**DOI:** 10.1093/jbmr/zjag009

**Published:** 2026-01-16

**Authors:** Lieve Verlinden, Sandra Calvo Blanco, Toke Liekens, Ingrid Stockmans, Karen Moermans, Dieter Schollaert, Chris Vercruysse, Tom Fiers, Tim Reyns, Nick Narinx, Tom De Waal, Leen Antonio, Ruslan I Dmitriev, Frank Claessens, Dirk Vanderschueren, Geert Carmeliet, Vanessa Dubois, Steve Stegen

**Affiliations:** Clinical and Experimental Endocrinology, Department of Chronic Diseases and Metabolism (CHROMETA), KU Leuven, 3000 Leuven, Belgium; Basic and Translational Endocrinology (BaTE), Department of Basic and Applied Medical Sciences, Faculty of Medicine and Health Sciences, Ghent University, 9000 Ghent, Belgium; Clinical and Experimental Endocrinology, Department of Chronic Diseases and Metabolism (CHROMETA), KU Leuven, 3000 Leuven, Belgium; Clinical and Experimental Endocrinology, Department of Chronic Diseases and Metabolism (CHROMETA), KU Leuven, 3000 Leuven, Belgium; Clinical and Experimental Endocrinology, Department of Chronic Diseases and Metabolism (CHROMETA), KU Leuven, 3000 Leuven, Belgium; Clinical and Experimental Endocrinology, Department of Chronic Diseases and Metabolism (CHROMETA), KU Leuven, 3000 Leuven, Belgium; Clinical Tissue and Cell Culture Core, Department of Human Structure and Repair, Faculty of Medicine and Health Sciences, Ghent University, 9000 Ghent, Belgium; Department of Clinical Biology, Ghent University Hospital, 9000 Ghent, Belgium; Department of Clinical Biology, Ghent University Hospital, 9000 Ghent, Belgium; Clinical and Experimental Endocrinology, Department of Chronic Diseases and Metabolism (CHROMETA), KU Leuven, 3000 Leuven, Belgium; Department of Laboratory Medicine, UZ Leuven, 3000 Leuven, Belgium; Clinical and Experimental Endocrinology, Department of Chronic Diseases and Metabolism (CHROMETA), KU Leuven, 3000 Leuven, Belgium; Department of Laboratory Medicine, UZ Leuven, 3000 Leuven, Belgium; Clinical and Experimental Endocrinology, Department of Chronic Diseases and Metabolism (CHROMETA), KU Leuven, 3000 Leuven, Belgium; Tissue Engineering and Biomaterials Group, Department of Human Structure and Repair, Faculty of Medicine and Health Sciences, Ghent University, 9000 Ghent, Belgium; Molecular Endocrinology Laboratory, Department of Cellular and Molecular Medicine, KU Leuven, 3000 Leuven, Belgium; Clinical and Experimental Endocrinology, Department of Chronic Diseases and Metabolism (CHROMETA), KU Leuven, 3000 Leuven, Belgium; Clinical and Experimental Endocrinology, Department of Chronic Diseases and Metabolism (CHROMETA), KU Leuven, 3000 Leuven, Belgium; Basic and Translational Endocrinology (BaTE), Department of Basic and Applied Medical Sciences, Faculty of Medicine and Health Sciences, Ghent University, 9000 Ghent, Belgium; Clinical and Experimental Endocrinology, Department of Chronic Diseases and Metabolism (CHROMETA), KU Leuven, 3000 Leuven, Belgium

**Keywords:** gender-affirming therapy, estrogen, bone, puberty suppression, transgender

## Abstract

Transgender individuals are increasingly seeking gender-affirming therapy. For children and adolescents, this typically involves puberty suppression followed by hormone treatment. However, potential long-term adverse effects on the growing skeleton remain poorly characterized, largely due to the lack of appropriate preclinical animal models. Existing models often rely on surgical puberty suppression and, in the case of estradiol (E2) administration, cause a high bone mass-phenotype that is not observed in the clinical setting. To address this, we developed a novel preclinical mouse model that mimics the medical approach used in transgirls and analyzed the effects of gender-affirming therapy on bone. Four-week-old male mice were treated with the gonadotropin-releasing hormone analogue degarelix (DGX) for pharmacological puberty suppression. After 4 wk, E2 was administered at different doses, and bone properties were analyzed after an additional 8 wk. DGX treatment effectively suppressed sex steroid signaling, leading to reduced bone mass and strength, which were dose-dependently restored by E2. While high E2 doses resulted in an excessive increase in bone mass, thereby precluding further mechanistic study, lower doses resulted in a bone phenotype resembling that of native females. At the cellular level, DGX-mediated puberty suppression resulted in increased bone resorption that exceeded bone formation, and also caused an accumulation of marrow adipocytes. Subsequent low-dose E2 administration reduced bone resorption, stimulated bone formation, and prevented the increase in bone marrow adiposity. In summary, we established and validated a mouse model that accurately mimics gender-affirming therapy initiated during early puberty and enables the study of its effects on bone.

## Introduction

Transgender persons experience a gender identity that differs from their designated sex at birth. The transgender population is rapidly expanding worldwide, with a growing number of individuals seeking medical care, including children and adolescents.^1^ The psychological burden that these people usually experience, referred to as gender dysphoria, is addressed by gender-affirming therapies, which may include puberty suppression as well as gender-affirming hormone (GAH) treatment and surgery. Specifically for transgender adolescents, puberty suppression by gonadotropin-releasing hormone analogue (GnRHa) administration is typically initiated at the earliest pubertal stages to halt further development of secondary sexual characteristics, and is generally followed by GAH treatment several years later.^2^ Consequently, transgender youth experience a relatively long period without exposure to sex steroids, although potential health risks are still poorly characterized.

Androgens and estrogens are crucial for bone development and maintenance by regulating the differentiation and function of different skeletal cell types, including bone-forming osteoblasts and osteocytes, bone-resorbing osteoclasts, as well as marrow adipocytes.^3^ Concerns have therefore been raised about potential adverse effects of prolonged GnRHa administration on the skeleton. Indeed, several studies have shown a negative effect of prolonged puberty suppression on bone mineral density, which can be restored, at least partially, following sex steroid administration.^4^ However, mechanistic insight is limited and long-term outcome data in adult transgender individuals who started their transition during early puberty are scarce. In this view, animal models provide a unique opportunity to address this significant gap of knowledge, facilitating further optimization of existing gender-affirming therapies.

Most preclinical models have focused on analyzing the impact of gender-affirming therapies on the adult skeleton, and thus did not investigate the effects of GnRHa administration.^5^ We therefore recently developed a mouse model that effectively mimics the clinical strategy for transboys, by treating peripubertal female mice with the GnRHa degarelix (DGX) to suppress puberty, followed by testosterone adminstration as GAH therapy.^6^ In contrast to this established Female-to-Male (FtM) model, most existing Male-to-Female (MtF) rodent models fail to adequately reflect the clinical situation. Indeed, surgical techniques like orchidectomy are often used for puberty suppression,^7^^,^^8^ although we have recently shown that this could also be achieved pharmacologically by GnRHa administration.^9^ More importantly, the relatively high estradiol (E2) doses that have been used result in massive bone gain,^7^^,^^8^^,^^10^ which is not observed in the clinical setting,^11^^,^^12^ likely because of interspecies differences in estrogen physiology.^13^^,^^14^

In this study, we therefore developed a clinically relevant mouse model that mimics the medical approach in transgirls, using GnRHa to suppress puberty and an optimized E2 regimen to avoid excessive bone mass accrual.

## Materials and methods

### Experimental setup of a murine adolescent MtF model

For puberty suppression,^9^ 4-wk-old male WT C57BL/6J mice were treated with the GnRHa degarelix (DGX; Firmagon, Ferring Pharmaceuticals) by subcutaneous injection (25 mg/kg body weight every 4 wk for a total duration of 12 wk),^9^ which was followed by E2 administration from 8 wk of age for a total duration of 8 wk. E2 was administered either through commercially available pellets (#E2-M/60, Belma Technologies, mean release of 1.8 μg E2/day; placebo pellets as control) or through a 1-cm silastic tube (Silclear Tubing, Degania Silicone), implanted on the back of the mice under isoflurane anesthesia. The silastic implants were filled with different doses of E2 (Sigma-Aldrich) diluted in cholesterol, ranging from 4 to 32 μg. The E2 release from a 1-cm stick filled with crystalline E2 is 3 μg E2/day, and shows a linear relationship with the length of the stick and the dilution factor.^15–17^ By calculation, the 4-32 μg dosing would correspond to a release of 1.5-12 ng E2/day, respectively. The following control groups were included: (1) age-matched female and male C57BL/6J mice, which were injected with vehicle (*aqua ad iniectabilia*) and received cholesterol-filled implants/placebo pellets; and (2) male DGX-treated mice that received cholesterol-filled implants.

Mice were group-housed in a conventional animal facility at 20 °C with 12 h light/dark cycle and ad libitum access to water and standard chow. To measure food intake, mice were kept in metabolic cages for 24 h. At 16 wk of age, body composition was analyzed and mice were euthanized by CO_2_ asphyxia, and serum and bones were collected for downstream analyses. All animal experiments were approved by the Institutional Animal Care and Research Advisory Committee of the KU Leuven (P093/2024).

### Mouse ovariectomy model

Ovariectomy (OVX) was performed in 8-wk-old female C57BL/6J mice as previously described^18^^,^^19^ and E2-releasing silastic sticks were implanted as detailed above. In sham-operated mice, ovaries were exposed but left intact. Sham-operated and OVX mice implanted with a cholesterol-filled stick were used as experimental controls. Eight weeks after surgery, body composition was analyzed and mice were sacrificed for bone phenotyping and serum analyses.

### EchoMRI

Total body fat mass and lean body mass were measured by quantitative magnetic resonance (EchoMRI-100H Analyzer, Echo Medical Systems) according to the manufacturer’s instructions.

### Serum measurements of sex hormones and bone turnover markers

Serum E2 levels were quantified by LC-MS/MS using the 6500+ triple-quadrupole mass spectrometer (AB Sciex) operating in the ESI- mode based on a previously described protocol^20^ with some adjustments. The 2D LC gradient was configured using a Xbridge protein (2.1 × 50 mm, 3.5 μm) BEH C4 column (Waters Corp) in the first dimension and a Kinetex (2.1 × 50 mm, 1.7 μm) biphenyl column (Phenomenex) in the second dimension. Mobile phases consisted of MeOH, H_2_O and 50 mM NH_4_F in H_2_O. Serum samples were extracted with 2.5 mL of diethylether after adding the internal standard (E2-d4). After mixing for 3 min, samples were frozen and decanted with supernatant collection. The supernatant was dried at 37 °C and reconstituted in 125 μL H_2_O/MeOH (45/55). A 100 μL aliquot was injected onto the LC-column. Measurements were acquired by the tandem mass spectrometer running in multiple reaction monitoring mode by exploiting transitions m/z 271 > 145 and 271 > 182 for E2 and 275 > 147 for the internal standard E2-d4. Data processing was performed using Sciex OS software. The limit of detection was 0.3 pg/mL.

Serum testosterone levels were quantified by LC-MS/MS using the Xevo TQ-XS IVD mass spectrometer coupled to an Acquity UPLC I-class (Waters Corp) equipped with an Acquity UPLC HSS T3 column (1.8 μm, 2.1 mm × 50 mm). Mobile phases consisted of MilliQ water and acetonitrile with post-column infusion of 2.5 mM ammonium fluoride solution at 20 μL/min. Samples (50 μL serum diluted in 250 μL internal standard solution) were incubated for 30 min at −20 °C to facilitate protein precipitation. Following centrifugation (10 min at  21 130 g), 250 μL of the supernatant was transferred to a 96-deepwell plate and subsequently dried under nitrogen flow. Evaporated samples were redissolved in 30% acetonitrile and incubated on a shaker for 5 min at 1000 rpm. After centrifugation, 10 μL of sample was injected into the LC-MS/MS system. Measurements were acquired in multiple reaction monitoring mode by exploiting transitions m/z 289.3 > 97.1 and 289.3 > 109.1 for testosterone and 292.2 > 100.0 for the internal standard (testosterone—^13^C_3_). Data processing was performed using TargetLynx software (Waters Corp). The limit of detection was 15 pg/mL.

Serum CTx-I and P1NP levels were measured using the RatLaps CTx-I EIA kit (#AC-06F1, Immunodiagnostic Systems) and the Rat/Mouse P1NP EIA kit (#AC-33F1, Immunodiagnostic Systems), respectively.

### Micro-CT

Micro-CT analysis of mineralized bone mass in tibiae was performed using the SkyScan 1272 system (Bruker) at a 5 μm pixel size, 0.5 mm aluminum filter, 60 kV, 83 μA, 180° angular rotation at 0.4° steps, and 3000 ms integration time. Projection data were reconstructed with the NRecon software (Bruker) and 3D morphometric parameters were calculated using CT Analyzer software (CTAn, Bruker) as previously described.^21–23^ For trabecular bone, the region between 0.75 and 2.25 mm distal of the growth plate was selected, and for cortical bone, the region between 4 and 4.5 mm. A threshold index of 100-255 for trabecular bone and 130-255 for cortical bone was used (global Otsu thresholding). Data were reported according to the guidelines of the American Society for Bone and Mineral Research.^24^ 3D image rendering was performed using the CT Visualization software (CTVol, Bruker).

### Biomechanical testing

To assess bone strength, a destructive 3-point bending test was performed on dissected femora using the LRXplus universal testing machine (Lloyd Instruments) at the BioAnalytical Tools and Technology (BATT) core facility (Ghent University) with a 100 N-load cell as described before.^25^ The femora were placed onto 2 supporting pins and the loading point was positioned at the middle of the femoral diaphysis. The loading pin was gradually moved downward with a speed of 0.1 mm/s until bone failure and the load-displacement curve was used to calculate ultimate load-to-failure, stiffness, and work-to-failure.

### Histology and histomorphometry

Histomorphometric analysis on tibiae was performed as described previously.^21^ Briefly, right tibiae collected at sacrifice were fixed overnight with 2% paraformaldehyde and subsequently decalcified in 0.5 M EDTA in PBS (pH 7.4) for two weeks at 4 °C. Decalcified bones were dehydrated in graded ethanol concentrations and embedded in paraffin. H&E-stained sections (4 μm thickness) were used to quantify osteoblasts and adipocytes, and tartrate-resistant acid phosphatase (TRAP) staining was used to quantify osteoclasts. Left tibiae were fixed overnight in Burckhardt solution and embedded in methyl-methacrylate (MMA). Unmineralized bone matrix was quantified on Goldner-stained MMA-embedded sections. To perform dynamic bone histomorphometry, mice were injected intraperitoneally with calcein (16 mg/kg body weight; Sigma-Aldrich) at day 4 and 1 prior to sacrifice.

Images were taken using an Axioscan 7 slide scanner microscope (Zeiss), and histomorphometric analysis was performed as described previously.^21^ Measurements were performed on at least two sections, each at least 40 μm apart. For each section, defined regions of interest were selected starting at 150 μm from the distal end of the growth plate. Data were expressed according to the American Society for Bone and Mineral Research standardized histomorphometry nomenclature.^26^

### Statistical analysis

Data are presented as box plots, showing the 25th to the 75th percentile with the median as a line and min to max as whiskers. All mice were randomly assigned to the different experimental groups and *n* represents the number of individually phenotyped mice. Statistical analysis was performed using GraphPad Prism v.10.2.3. To compare the different experimental groups, one-way analysis of variance (ANOVA) and Tukey’s multiple comparisons test were performed. Differences were considered as statistically significant when *p* < .05.

## Results

### E2 administration through commercially available pellets leads to abberant bone mass accrual in DGX-treated male mice

**Figure 1:**
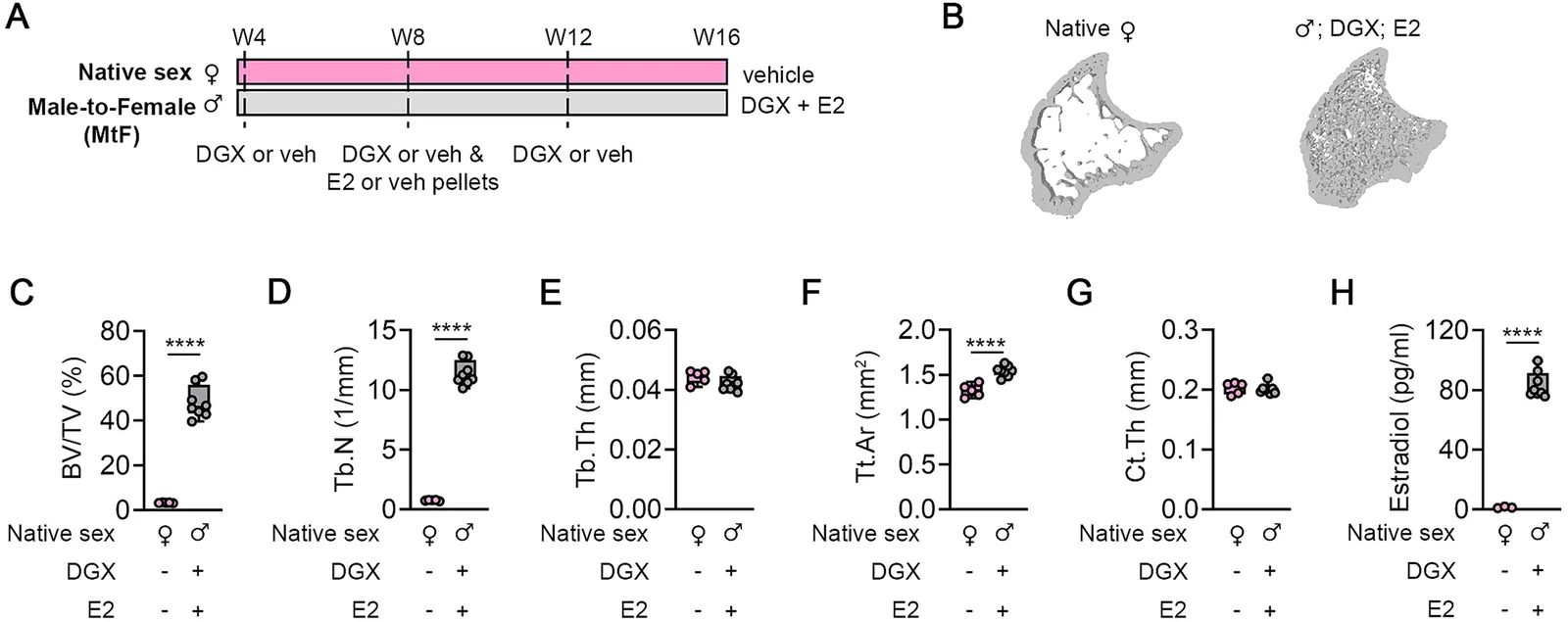
Administration of estradiol (E2) via commercially available pellets leads to aberrant bone formation. (A) Schematic representation of the experimental setup. Starting at 4 wk of age, degarelix (DGX) or vehicle (veh) was administered via subcutaneous injections every 4 wk. In addition, from 8 wk of age, mice received E2 or vehicle delivered via subcutaneously implanted commercially available pellets. Mice were euthanized at the age of 16 wk. (B) 3D micro-CT models of the tibial metaphysis of 16-wk-old mice subjected to the protocol described in panel A. A representative image from each experimental group is shown. (C-E) Micro-CT analysis of trabecular bone parameters of the tibial metaphysis, showing quantification of trabecular bone volume fraction (BV/TV; C), trabecular number (Tb.N; D), and trabecular thickness (Tb.Th; E) in 16-wk-old mice (*n* = 5-6/group). (F and G) Micro-CT analysis of cortical bone parameters of the tibial diaphysis, showing quantification of the total cross-sectional tissue area (Tt.Ar; F) and cortical thickness (Ct.Th; G) in 16-wk-old mice (*n* = 5–6/group). (H) Serum estradiol levels in 16-wk-old mice (*n* = 3-8/group). Data are presented as box plots, showing the 25th to the 75th percentile with the median as a line and min to max as whiskers. ^****^p < .0001 (one-way ANOVA with Tukey’s post-hoc test).

Currently available MtF models use surgical castration to suppress puberty and high doses of E2 that cause a massive increase in bone mass.^7^^,^^8^ To develop a more clinically relevant mouse model, we therefore treated 4-wk-old male mice with the GnRHa DGX, which is known to adequately block sexual maturation,^9^ and implanted commercially available E2-releasing pellets at 8 wk of age for an additional 8 wk-period (Figure 1A). This approach resulted in substantially higher trabecular bone volume and total cross-sectional tissue area without affecting cortical thickness (Figure 1B-G and Figure S1A-F). The profound induction in bone mass accrual is likely related to the undesirable high circulating E2 levels (Figure 1H) and indicates that careful E2 titration is necessary.

### Effect of E2 dosing on hormone-responsive tissues and body composition in DGX-treated male mice

**Figure 2:**
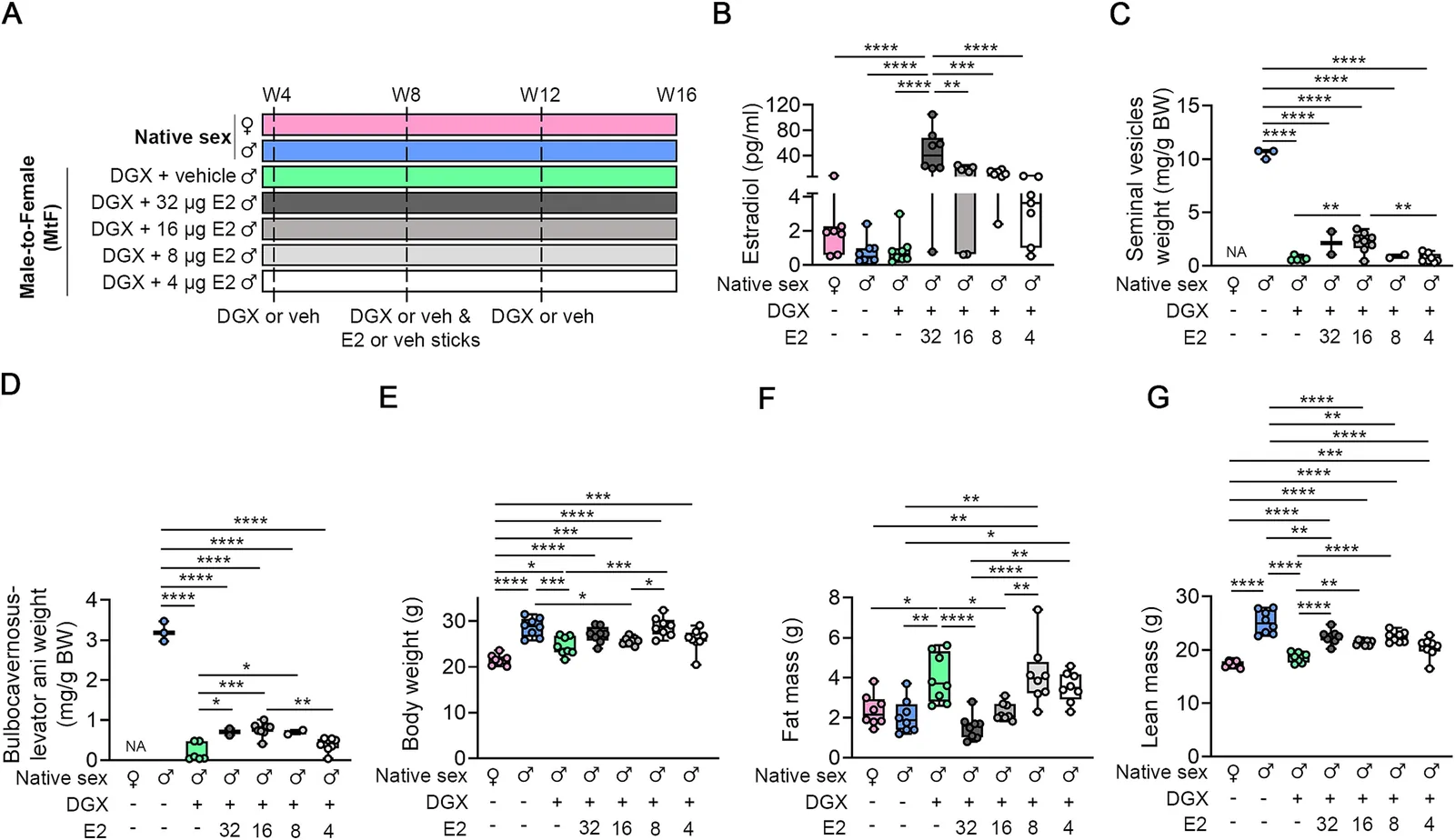
Experimental design and validation of the Male-to-Female mouse model. (A) Schematic representation of the experimental setup. Starting at 4 wk of age, degarelix (DGX) or vehicle (veh) was administered via subcutaneous injections every 4 wk. In addition, from 8 wk of age, mice received estradiol (E2) or vehicle delivered via subcutaneously implanted silastic sticks. Mice were euthanized at the age of 16 wk. (B) Serum estradiol levels in 16-wk-old mice (*n* = 7-8/group). (C and D) Wet weight of the seminal vesicles (C) and the bulbocavernosus-levator ani complex (D), expressed relative to total body weight (BW), in 16-wk-old mice subjected to the protocol as described in panel A (*n* = 2-8/group) (NA, not applicable). (E-G) Body weight (E), total body fat mass (F), and lean body mass (G) in 16-wk-old mice (*n* = 8/group). Data are presented as box plots, showing the 25th to the 75th percentile with the median as a line and min to max as whiskers. ^*^p < .05, ^**^p < .01, ^***^p < .001, ^****^p < .0001 (one-way ANOVA with Tukey’s post-hoc test).

To address the E2-mediated bone overshoot, we opted to perform a dosing experiment by implanting silastic sticks containing 32-4 μg of E2 in puberty-suppressed male mice. Untreated male and female mice were used as controls for native and intended sex, respectively. A schematic of the experimental setup is shown in Figure 2A. Compared to female controls, we observed that administration of the 32-μg E2 stick resulted in 20-fold higher serum E2 levels, which dose-dependently dropped with 16 or 8 μg E2, although E2 levels were still elevated 5-to-6-fold (Figure 2B). In mice that received sticks containing 4 μg E2, circulating E2 levels were more close to those observed in female control mice (Figure 2B). DGX treatment resulted in slightly lower circulating E2 levels (Figure 2B), although these values were close to or below the limit of detection, thereby precluding to correctly quantify the reduction. Serum testosterone levels were below the limit of detection in all DGX-treatment conditions, independent of E2 supplementation (Figure S2A).

We first confirmed the known action of DGX and E2 administration on androgen-sensitive organs and body composition. As expected from our previous work,^9^ the weights of the seminal vesicles and the bulbocavernosus-levator ani muscle complex were manifestly lower in DGX-treated mice (Figure 2C and D). Upon E2 administration to DGX-treated mice, the weight of both tissues was slightly higher, albeit well-below the control male level (Figure 2C and D). A similar effect of DGX treatment was observed on testis and kidney weight, whereas their weight was not altered upon E2 administration (Figure S2B and C). In addition to the changes in sex steroid-sensitive tissues, body weight of DGX-treated male mice was significantly lower, with only a marginal effect of additional E2 administration (Figure 2E).

Body composition was more profoundly altered by GnRHa and GAH therapy, as evidenced by EchoMRI analysis (Figure 2F and G and Figure S2D and E). DGX treatment resulted in higher total body fat mass and lower lean body mass. Regarding E2, a dose-dependent decrease in adiposity was observed compared to mice treated with DGX alone, while lean mass was modestly increased. Of note, animals treated with the higher E2 doses (ie, 32 and 16 μg E2) displayed a male-like pattern of body composition, whereas the fat and lean mass of the mice receiving the lower E2 doses (ie, 8 and 4 μg E2) were similar to those of the female controls (Figure 2F and G). The observed changes in body composition were not caused by E2-mediated changes in food intake (Figure S2F).

Taken together, our data indicate that treating peripubertal male mice with DGX effectively suppresses sex steroid signaling, and subsequent administration of a low E2 dose results in a body composition resembling that of native females.

### Skeletal effects of E2 dosing in DGX-treated male mice

**Figure 3:**
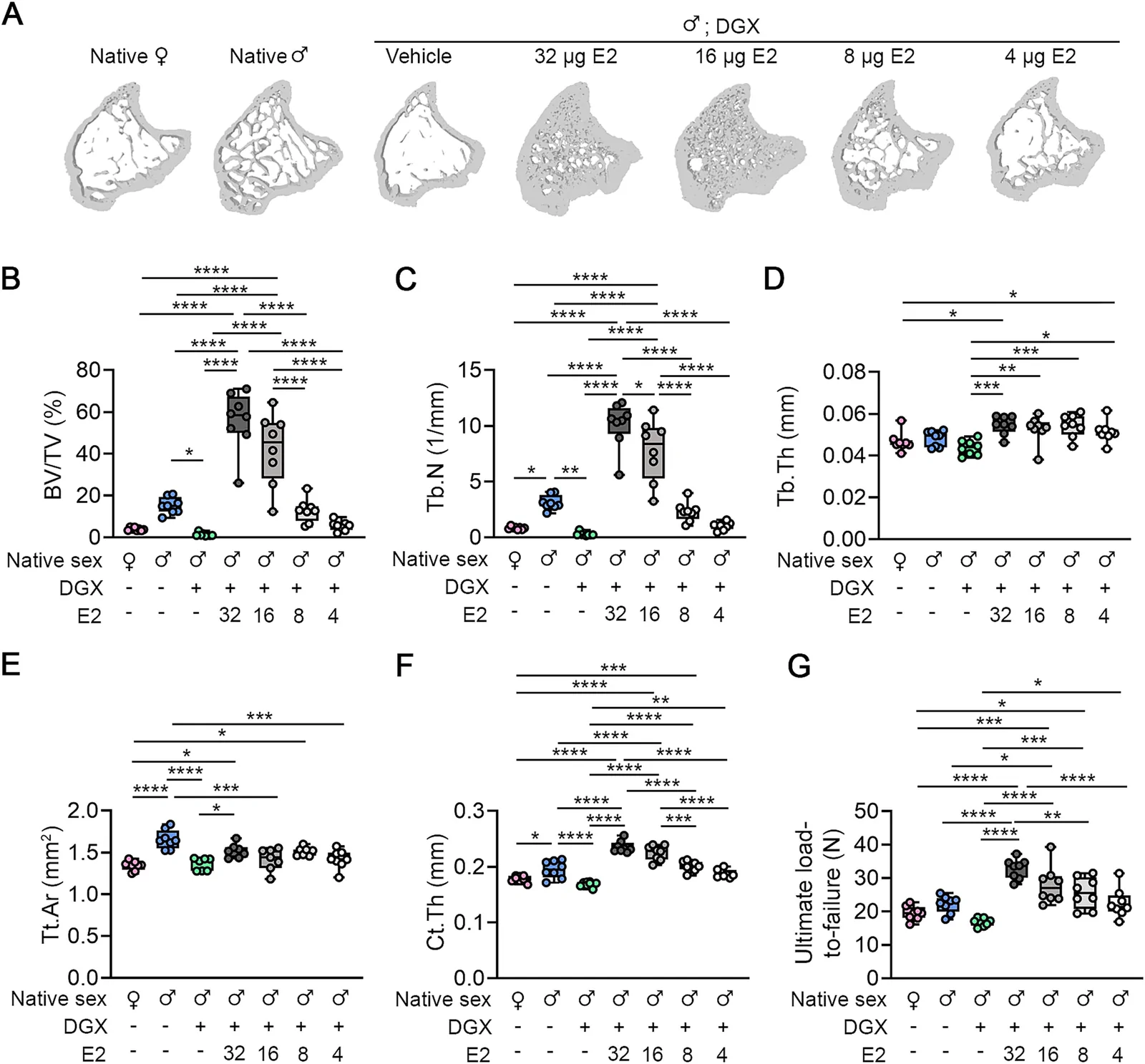
A low E2 dosing regimen is required to avoid massive bone gain in DGX-treated male mice. (A) 3D micro-CT models of the tibial metaphysis of 16-wk-old mice subjected to the protocol described in Figure 2A. A representative image from each experimental group is shown. (B-D) Micro-CT analysis of trabecular bone parameters of the tibial metaphysis in 16-wk-old mice, showing quantification of trabecular bone volume fraction (BV/TV; B), trabecular number (Tb.N; C), and trabecular thickness (Tb.Th; D) (*n* = 8/group). (E and F) Micro-CT analysis of cortical bone parameters of the tibial diaphysis of 16-wk-old mice, showing quantification of the total cross-sectional tissue area (Tt.Ar; E) and cortical thickness (Ct.Th; F) (*n* = 8/group). (G) Ultimate load-to-failure, as determined by three-point bending, of femurs from 16-wk-old mice (*n* = 8/group). Data are presented as box plots, showing the 25th to the 75th percentile with the median as a line and min to max as whiskers. ^*^p < .05, ^**^p < .01, ^***^p < .001, ^****^p < .0001 (one-way ANOVA with Tukey’s post-hoc test).

We next assessed the skeletal effects of the different E2 doses, aiming to identify an E2 treatment regimen that restores the negative effects of puberty suppression without causing a manifest increase in bone mass, which would better align with the clinical situation.^11^^,^^12^ While tibia length was not affected by either DGX or E2 treatment (Figure S3A), we observed significant changes in mineralized bone mass. As expected from our previous work,^9^ treating peripubertal male mice with DGX resulted in significantly lower trabecular bone mass, and E2 administration dose-dependently increased this parameter, reaching values up to 3-fold higher than those in male control mice (Figure 3A and B). The higher bone mass in E2-treated mice was mainly due to a higher trabecular number, accompanied by lower trabecular separation, whereas trabecular thickness was only modestly affected (Figure 3C and D and Figure S3B). In contrast to the severe high bone mass-phenotype induced by the higher doses of E2 (ie, 32 and 16 μg E2), mice that were implanted with sticks containing the lower E2 doses (ie, 8 and 4 μg E2) displayed trabecular bone parameters that ranged between those of male and female controls (Figure 3A and B).

Hormonal manipulation in peripubertal male mice also affected cortical bone parameters. Total cross-sectional tissue area was reduced in DGX-treated mice, and only elevated when mice were treated with 32 μg E2 (Figure 3E). In contrast, cortical thickness in DGX-treated animals was dose-dependently increased upon E2 supplementation, which correlated with a decrease in marrow area and was likely caused by a relative expansion at the endocortical side (Figure 3F and Figure S3C-G). Compared to male and female controls, only the lower E2 doses (ie, 8 and 4 μg E2) did not result in increased cortical thickness (Figure 3F).

We also determined whether the changes in cortical bone mass were associated with altered mechanical properties, by performing a 3-point bending test. In line with the micro-CT data, we observed that the ultimate load-to-failure and work-to failure in DGX-treated mice tended to be lower than in male controls, suggesting reduced bone strength (Figure 3G and Figure S3H-J). E2 administration dose-dependently increased bone strength, with the lower E2 doses yielding ultimate load-to-failure values in the male control range (Figure 3G). Bone stiffness did not differ significantly among the different experimental conditions (Figure S3K). The fact that low-dose E2 administration did not result in excessive bone mass or strength was likely related to the low circulating E2 levels (Figure 2B).

Taken together, these data show that GnRHa therapy initiated at the peripubertal stage in male mice manifestly impairs bone properties. E2 administration can restore the bone defects, but should be carefully titrated to avoid abberant bone mass accrual.

### Effects of low-dose E2 administration on bone-residing cell types in DGX-treated male mice

**Figure 4:**
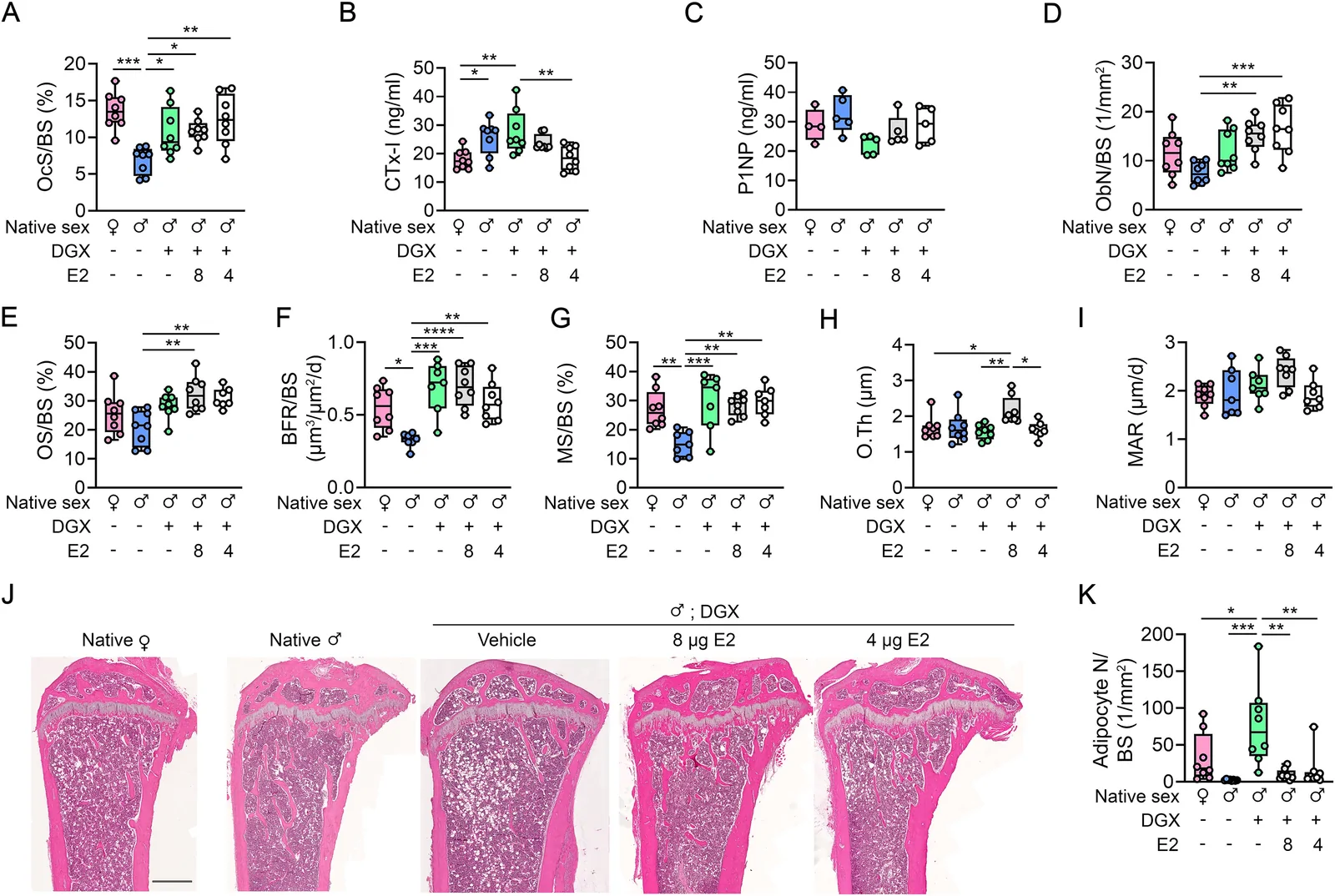
Effect of DGX and E2 treatment on bone-residing cell types. (A) Quantification of osteoclast surface per bone surface (OcS/BS), assessed on TRAP stained-sections of the tibial metaphysis from 16-wk-old mice subjected to the protocol described in Figure 2A (*n* = 8/group). (B and C) Serum CTx-I levels (B; *n* = 8/group) and serum P1NP levels (C; *n* = 4-5/group) in 16-wk-old mice. (D) Quantification of osteoblast number per bone surface (ObN/BS), assessed on H&E stained-sections of the tibial metaphysis from 16-wk-old mice (*n* = 8/group). (E) Quantification of osteoid surface (OS/BS) on Goldner-stained sections of the tibial metaphysis from 16-wk-old mice (*n* = 8/group). (F) Quantification of bone formation rate (BFR) after analysis of calcein labels in the tibial metaphysis of 16-wk-old mice (*n* = 8/group). (G) Quantification of mineralizing surface (MS/BS) after analysis of calcein labels in the tibial metaphysis of 16-wk-old mice (*n* = 8/group). (H) Analysis of osteoid thickness (O.Th) on Goldner-stained sections of the tibial metaphysis from 16-wk-old mice (*n* = 8/group). (I) Quantification of mineral apposition rate (MAR) after analysis of calcein labels in the tibial metaphysis of 16-wk-old mice (*n* = 8/group). (J and K) H&E staining of the tibial metaphysis from 16-wk-old mice (J) with quantification of adipocyte number per bone surface (adipocyte N/BS; K) (*n* = 8/group). Scale bar is 500 μm. Data are presented as box plots, showing the 25th to the 75th percentile with the median as a line and min to max as whiskers. ^*^p < .05, ^**^p < .01, ^***^p < .001, ^****^p < .0001 (one-way ANOVA with Tukey’s post-hoc test).

Based on the observation that low-dose E2 administration in puberty-suppressed male mice does not cause abberant bone gain, we selected these doses for further bone phenotyping using histology and serum analysis. Histomorphometric analysis of TRAP-stained sections revealed a higher osteoclast surface in DGX-treated mice compared to control male mice (Figure 4A). This effect was however not reflected in similar changes in serum collagen type I cross-linked C-telopeptide (CTx-I) levels (Figure 4B), although CTx-I levels were relatively highest in DGX-treated mice, suggesting more bone resorption in these conditions. On the other hand, administration of E2 in DGX-treated mice did not manifestly affect osteoclast surface but tended to dose-dependently decrease serum CTx-I levels (Figure 4A and B), suggesting that bone resorption might be reduced.

We next investigated the effect of DGX and E2 treatment on bone formation parameters. Although serum procollagen 1 N-terminal propeptide (P1NP) levels did not differ significantly between groups (Figure 4C), histomorphometric analysis of H&E-stained sections revealed that the number of cuboidal osteoblasts spanning the bone surface tended to be higher in DGX-treated mice and was significantly higher in E2-treated mice (Figure 4D). When investigating extracellular matrix production using Goldner staining and matrix mineralization by calcein incorporation, we observed that osteoid surface and bone formation rate were higher in DGX-treated mice than in native males, and were only mildly affected upon E2 administration (Figure 4E and F). These changes mostly related to the higher number of mineralizing osteoblasts spanning the bone (Figure 4G), as osteoid thickness and mineral apposition rate were generally not affected by either DGX or E2 treatment (Figure 4H and I).

Finally, we studied the impact of gender-affirming therapy on bone marrow adipose tissue, which is known to regulate bone remodeling by promoting bone resorption and blocking bone formation.^27^ Similar to our previous study using female mice,^6^ prolonged puberty suppression in male mice resulted in a higher bone marrow adiposity, as evidenced by the 30-fold higher adipocyte number upon DGX administration (Figure 4J and K). The number of bone marrow adipocytes was significantly lower in E2-treated mice than in control males, irrespective of its dose (Figure 4J and K).

Taken together, we demonstrate that prolonged puberty suppression in male mice results in a high bone turnover bone loss phenotype, as evidenced by higher bone resorption and formation parameters, and causes a higher bone marrow adiposity. However, bone resorption likely exceeds bone formation, resulting in net bone loss. Subsequent low-dose E2 administration in DGX-treated mice shifts this balance by yielding a higher number of bone-forming osteoblasts and lower osteoclast activity, while simultaneously preventing the DGX-associated elevation in the number of bone marrow adipocytes.

### Validation of the low-dose E2 regimen using an OVX model

**Figure 5:**
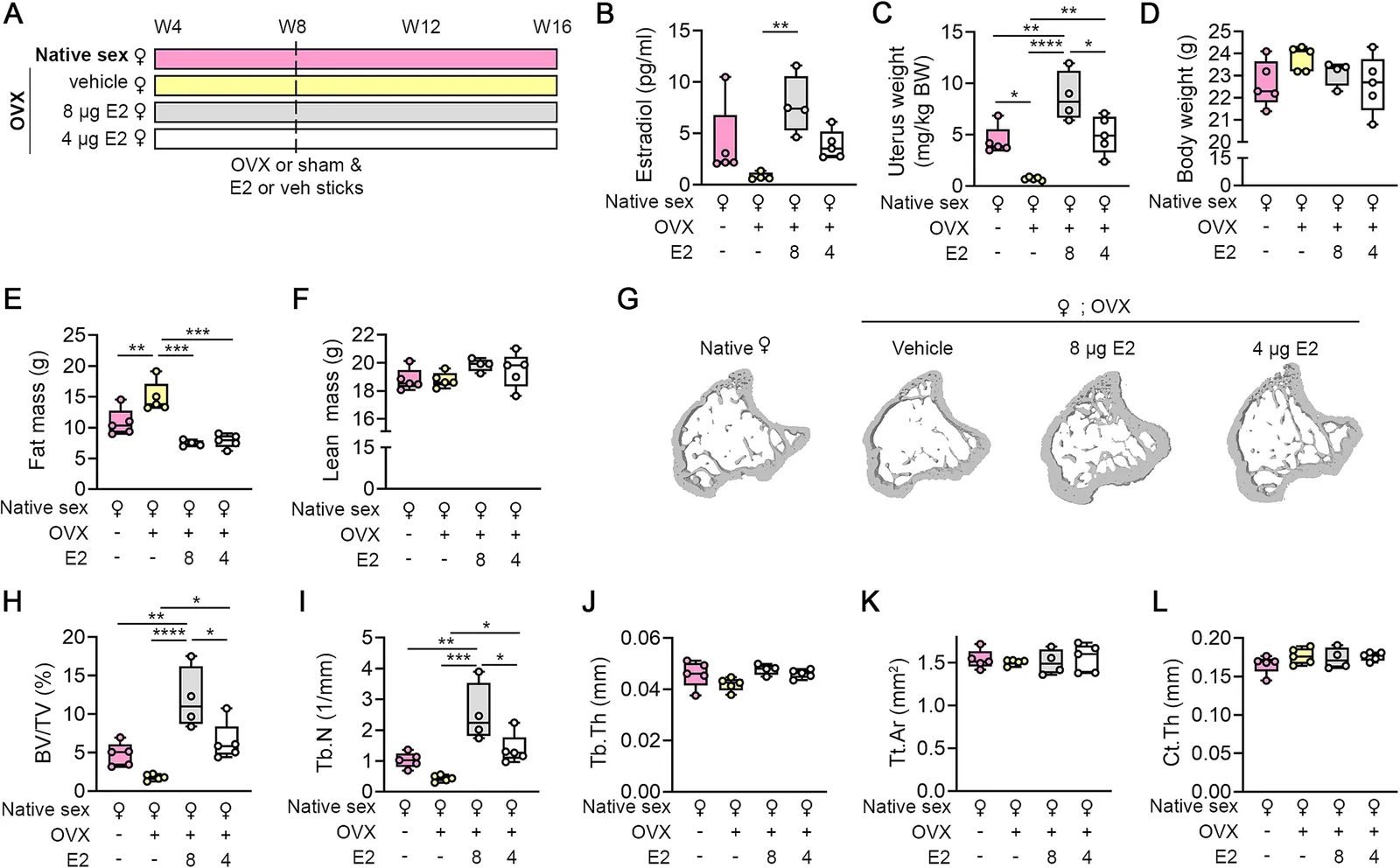
Validation of the selected E2 doses using a mouse ovariectomy (OVX) model. (A) Schematic representation of the experimental setup. Eight-week-old female mice were ovariectomized or sham-operated and received estradiol (E2) or vehicle via subcutaneously implanted silastic sticks. Mice were euthanized at the age of 16 wk. (B) Serum estradiol levels in 16-wk-old mice (*n* = 4-5/group). (C) Uterus wet weight, expressed relative to body weight (BW), of 16-wk-old mice subjected to the protocol described in panel A (*n* = 4-5/group). (D-F) Body weight (D), total body fat mass (E) and lean body mass (F) of 16-wk-old mice (*n* = 4-5/group). (G) 3D micro-CT models of the tibial metaphysis of 16-wk-old mice. A representative image from each experimental group is shown. (H-J) Micro-CT analysis of trabecular bone parameters of the tibial metaphysis of 16-wk-old mice, showing quantification of trabecular bone volume fraction (BV/TV; H), trabecular number (Tb.N; I), and trabecular thickness (Tb.Th; J) (*n* = 4-5/group). (K and L) Micro-CT analysis of cortical bone parameters of the tibial diaphysis of 16-wk-old mice, showing quantification of the total cross-sectional tissue area (Tt.Ar; K) and cortical thickness (Ct.Th; L) (*n* = 4-5/group). Data are presented as box plots, showing the 25th to the 75th percentile with the median as a line and min to max as whiskers. ^*^p < .05, ^**^p < .01, ^***^p < .001, ^****^p < .0001 (one-way ANOVA with Tukey’s post-hoc test).

Our data indicate that a low-dose E2 treatment in DGX-treated male mice results in a more physiologically and clinically relevant response in terms of body composition and bone parameters. For final validation, we tested the 8 and 4 μg E2 doses using a mouse OVX model (Figure 5A), in which endogenous estrogen production is known to be lower, as evidenced by the lower serum E2 levels and uterus weight (Figure 5B and C). Administration of 4 μg E2 restored these parameters to the level of sham-operated mice, whereas we observed higher serum E2 levels and uterus weight when OVX mice were treated with 8 μg E2 (Figure 5B and C). As expected from disturbed estrogen homeostasis,^28^ OVX tended to display a higher body weight and total body fat mass, effects that were counteracted to the same extent by both E2 doses (Figure 5D-F and Figure S4A and B).

Concerning bone properties, we observed that E2 administration prevented the lower trabecular bone volume observed in OVX mice (Figure 5G and H), which was mainly attributable to a higher trabecular number without affecting trabecular thickness (Figure 5I and J and Figure S4C). Importantly, while 4 μg E2 restored trabecular bone parameters to those observed in sham-operated mice, a dose of 8 μg E2 resulted in trabecular bone mass and trabecular number that were approximately 3-fold higher than in sham controls, which is in line with the elevated serum E2 levels (Figure 5B and H). Cortical bone parameters were not manifestly affected by OVX nor by E2 administration (Figure 5K and L, Figure S4D-H), although the ultimate load-to-failure was slightly higher with the 8 μg E2 dose (Figure S4I-M).

Taken together, our data indicate that low-dose E2 administration effectively counteracts the OVX-mediated effects on bone and body composition. However, the 8 μg E2 dose still caused a modest supraphysiological effect on bone, and we therefore propose the 4 μg E2 dose as the preferred E2 regimen when studying the effects of puberty suppression and GAH therapy in peripubertal male mice.

## Discussion

**Figure 6:**
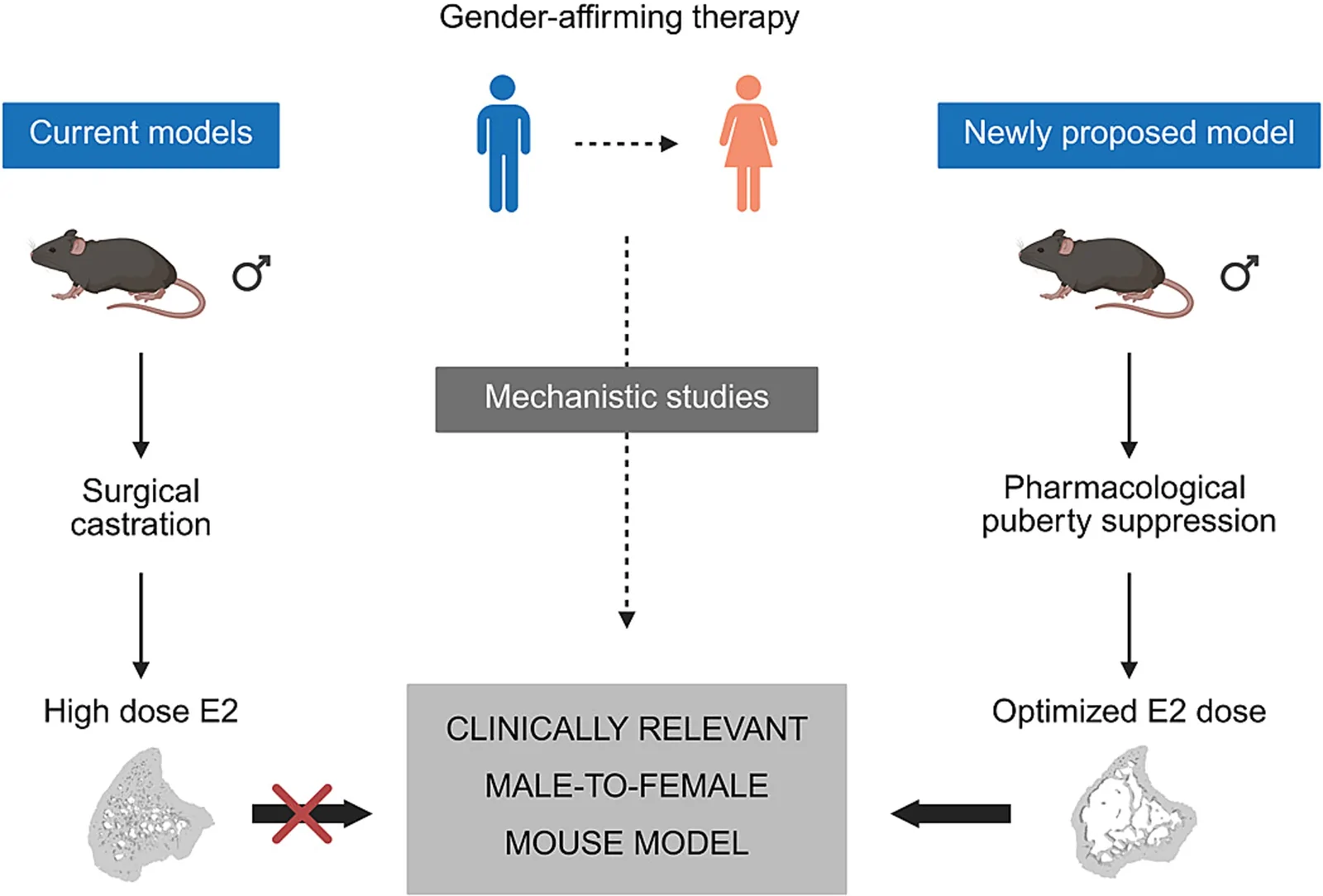
Schematic representation of research findings. Currently available murine Male-to-Female (MtF) models rely on surgical castration for puberty suppression and display overt estradiol (E2)-mediated bone gain, which does not mirror the clinical situation and precludes further mechanistic studies. Here, we propose a novel MtF model, using degarelix (DGX) to suppress puberty together with an optimized low-dose E2 treatment regimen to avoid aberrant bone formation.

Currently available mouse models do not adequately mimic the clinical approach used in transgirls, as they rely on surgical castration for puberty suppression, and the relatively high administered E2 doses cause an unfavorable high bone mass.^7^^,^^8^^,^^11^^,^^12^ These technical limitations hinder proper mechanistic studies, and we therefore developed a clinically relevant MtF mouse model by using a GnRHa to pharmacologically suppress puberty followed by carefully titrated E2 administration that avoids excessive bone gain (Figure 6).

Setting up appropriate preclinical animal models can be technically challenging, but is however critical to understand disease mechanisms and to develop novel therapeutic approaches. When studying potential skeletal effects of gender-affirming therapy in transgirls, orchidectomy is often performed in rodents to achieve puberty suppression, but this surgical procedure lacks clinical translatability. Moreover, these surgeries may cause animal distress, which may confound experimental outcome.^29^ Our pharmacological approach therefore also contributes to the refinement of rodent transgender studies. Finally, unlike the GnRHa-mediated puberty suppression used in both our study and in the clinical setting,^1^^,^^2^ orchidectomy results in elevated, rather than suppressed, gonadotropin levels,^30^^,^^31^ which may differentially affect bone cells^32^ and thereby complicate comparing skeletal outcomes. Here, we provide strong experimental evidence that DGX treatment effectively suppresses puberty in developing male mice, and thus better reflects the clinical practice. Another caveat of currently used rodent MtF models is the excessive bone mass observed after administration of supraphysiological E2 doses,^7^^,^^8^^,^^10^ that is, surpassing the bone volume in native females and even that of native males. These effects contrast with the overall minor effects observed in transfeminine adolescents treated with the standard E2 dosing regimen.^11^^,^^12^ Based on our dose-optimization and validation study using ovariectomized mice, we propose the lowest E2 dose we tested, that is, 4 μg, as being appropriate to study the effects of GAH therapy on bone. Although lower doses were not tested in this study due to animal-number constraints, we show that this specific E2 dose yields circulating E2 levels that fall within the reported physiological range in female mice^33^ and results in a bone phenotype that is reminiscent to that of transfeminine adolescents.^11^^,^^12^

This clinically more relevant MtF mouse model allowed us to analyze the effect of gender-affirming therapy on bone properties. While bone length was not affected, which is in accordance with recent studies in transfemale mice and adolescents,^8^^,^^34^ we observed that DGX treatment and E2 administration differentially impacted mineralized bone mass. Indeed, in line with previous findings using castration-based MtF models and in transadolescents receiving GnRHa treatment,^7^^,^^8^^,^^35^ prolonged DGX-mediated puberty suppression in peripubertal male mice resulted in a low trabecular and cortical bone mass, which was associated with reduced bone strength. These bone defects were restored to the level of native female mice upon low-dose E2 administration, thereby mirroring clinical observations^36^ and thus further supporting the translational potential of our proposed MtF model. Intriguingly, although E2 treatment can prevent GnRHa-mediated bone loss, two recent clinical studies have reported that transwomen who received GAH during adulthood show an increased fracture risk compared to cismen.^37^^,^^38^ While the underlying mechanisms are unclear, and might be related to the relatively low baseline bone density in transfeminine individuals, reduced physical activity or other lifestyle-related factors,^11^^,^^39^ these findings suggest that gender-affirming therapy started in early life may prime bone for future insults, although this hypothesis requires further investigation.

Using histological analyses and serum bone turnover markers, we noted that puberty suppression resulted in an increased trend in both bone resorption and formation. However, the resorptive activity likely outweighed formation as we observed net bone loss, which is in line with studies using castrated mice^40^ and observations in DGX-treated men in the context of androgen deprivation therapy.^41^^,^^42^ Interestingly, in these clinical studies, it was shown that bone formation may also be negatively affected early after DGX administration.^42^ Whether these early dynamic changes in bone (re)modeling also occur in transgirls could therefore be further studied using our newly developed MtF mouse model. The DGX-induced bone loss was rescued by E2 administration, which is in accordance with the known skeletal effects of E2,^3^ although interspecies differences might exist, especially at higher doses.^14^ These data however contrast with a recently published study describing an adolescent MtF model using high-dose E2 administration, which increased both bone resorption and formation.^8^ These effects are likely caused by the uncoupling of bone remodeling due to the supraphysiological E2 levels, thereby reinforcing the rationale for the careful E2 titration performed in this study. In addition to the changes in osteoblast and osteoclast behavior, we observed that puberty suppression and GAH therapy caused manifest changes in bone marrow adiposity, which is in accordance with our recent study using FtM mice and clinical observations in GnRHa-treated transgender youth.^6^^,^^43^ Interestingly, sex steroids regulate the differentiation of bone marrow progenitors into adipocytes,^44^ which, in turn, inhibit bone formation while stimulating bone resorption.^45^ However, whether a similar mechanism explains the changes in bone mass observed upon gender-affirming therapy warrants further investigation. Since inherent differences in bone properties and the cellular composition of the bone microenvironment have been observed between male and female mice, future studies should include dynamic phenotyping to characterize the effects of puberty suppression and hormone supplementation, focusing on earlier timepoints. Additionally, the effects of gender-affirming therapy in older mice, thereby modeling young adults, warrants further investigation.

Our newly established MtF mouse model also allows for the investigation of phenotypic alterations caused by gender-affirming therapy beyond its effects on the skeletal system. For example, our data indicate that GnRHa administration to peripubertal male mice leads to marked alterations in body composition, mainly characterized by a high fat mass, which is again restored upon low-dose E2 treatment. These findings align with previous findings using castration-based rodent models^16^^,^^46^ and clinical studies in transgirls,^35^^,^^47^ further emphasizing the clinical translatability of our MtF model and its potential use to study the impact of gender-affirming therapy on whole-body physiology and tissue function. Of note, the observation that E2 administration only restores certain physiological traits, and not others, may be explained by the fact that parameters, such as fat mass and bone properties, mainly rely on aromatization of testosterone, which is not the case for traits that are primarily regulated by androgenic signaling, such as lean mass and muscle weight.^48^^,^^49^ In addition, sex chromosome complement may further shape these outcomes, as studies using the 4-core genotypes mouse model revealed that although gonadal sex has a major role, sex chromosomes also contribute to musculoskeletal mass and bone strength.^50^

In conclusion, our work established a clinically relevant mouse model that more closely reflects the endocrine changes experienced by transgirls undergoing puberty suppression followed by estrogen treatment. By optimizing the E2 dosing regimen, we demonstrate that the GnRHa-induced bone defects can be effectively mitigated with low-dose E2, without causing excessive bone mass accrual. The partial restoration of some physiological traits in our model underscores that estrogen treatment cannot fully substitute for the combined estrogenic, androgenic and sex chromosome-dependent contributions that shape a male phenotype. Nevertheless, this model provides a reliable tool to further investigate the potential long-term adverse skeletal effects of hormonal manipulation in children and young adolescents, thereby contributing to the optimization of gender-affirming care and the maintenance of bone health throughout life.

## Supplementary Material

verlinden_supplemental_figures_legends_zjag009

## Data Availability

All data generated during this study have been included in this manuscript. All other information required to reproduce our findings is available from the corresponding author upon reasonable request.
