# Supplementary material for: A novel model to study the impact of gender-affirming therapy on bone in young male mice
Source: J Bone Miner Res. 2026 Jan 16;41(9):1027–38. doi: 10.1093/jbmr/zjag009 (PMC13525205; doi:10.1093/jbmr/zjag009)
Supplement: Supplementary file 1 [file verlinden_supplemental_figures_legends_zjag009.docx]

**SUPPLEMENTAL FIGURES**

**Supplemental Figure S1. Effect of estradiol administration through commercially available pellets on bone parameters in DGX-treated male mice. (A-F)** MicroCT-based quantification of trabecular separation (Tb.Sp; **A**), cortical bone area (Ct.Ar; **B**), cortical area fraction (Ct.Ar/Tt.Ar; **C**), medullary area (Ma.Ar; **D**), periosteal perimeter (Ps.Pm; **E**), and endocortical perimeter (Ec.Pm; **F**) in tibiae from 16-week-old mice subjected to the protocol described in Figure 1A (n=5-6/group). Data are presented as box plots, showing the 25th to the 75th percentile with the median as a line and min to max as whiskers. ***p<0.001, ****p<0.0001 (one-way ANOVA with Tukey’s post-hoc test).

**Supplemental Figure S2. Effect of DGX and E2 treatment on testosterone levels, organ weight, body composition and food intake.** **(A)** Serum testosterone levels in 16-week-old mice subjected to the protocol described in Figure 2A (n=4-5/group) (ND = not detected)**. (B-C)** Wet weight of testis **(B)** and kidney **(C)**, expressed relative to total body weight (BW), of 16-week-old mice (n=2-8/group). **(D-E)** Total body fat mass **(D)** and lean body mass **(E)**, expressed as percentage of total body weight, of 16-week-old mice (n=8/group) **(F)** Food intake of 16-week-old mice after housing in individual metabolic cages for 24 hours (n=2-8/group). Data are presented as box plots, showing the 25th to the 75th percentile with the median as a line and min to max as whiskers. *p<0.05, ****p<0.0001 (one-way ANOVA with Tukey’s post-hoc test).

**Supplemental Figure S3.** **Effect of DGX and E2 treatment on bone parameters. (A)** Tibia length of 16-week-old mice subjected to the protocol described in Figure 2A. **(B-G)** MicroCT-based quantification of trabecular separation (Tb.Sp; **B**), cortical bone area (Ct.Ar; **C**), cortical area fraction (Ct.Ar/Tt.Ar; **D**), medullary area (Ma.Ar; **E**), periosteal perimeter (Ps.Pm; **F**), and endocortical perimeter (Ec.Pm; **G**) in tibiae from 16-week-old mice (n=8/group). **(H-I)** Length **(H)** and cross-sectional area at mid-length **(I)** of femurs subjected to three-point bending. **(J-K)** Work-to-failure **(J)** and stiffness **(K)** , as determined by three-point bending, of femurs from 16-week-old mice (n=8/group). Data are presented as box plots, showing the 25th to the 75th percentile with the median as a line and min to max as whiskers. *p<0.05, **p<0.01, ***p<0.001, ****p<0.0001 (one-way ANOVA with Tukey’s post-hoc test).

**Supplemental Figure S4. Effect of low-dose E2 administration on bone properties in ovariectomized (OVX) mice. (A-B)** Total body fat mass **(A)** and lean body mass **(B)**, expressed as percentage of body weight, of 16-week-old mice subjected to the protocol described in Figure 5A (n=4-5/group). **(C-H)** MicroCT-based quantification of trabecular separation (Tb.Sp; **C**), cortical bone area (Ct.Ar; **D**), cortical area fraction (Ct.Ar/Tt.Ar; **E**), medullary area (Ma.Ar; **F**), periosteal perimeter (Ps.Pm; **G**), and endocortical perimeter (Ec.Pm; **H**) in tibiae from 16-week-old mice. **(I-J)** Length **(I)** and cross-sectional area at mid-length **(J)** of femurs subjected to three-point bending. **(K-M)** Ultimate load-to-failure **(K)**, work-to-failure **(L)**, and stiffness **(M)** as determined by three-point bending, of femurs from 16-week-old mice (n=4-5/group). Data are presented as box plots, showing the 25th to the 75th percentile with the median as a line and min to max as whiskers. *p<0.05, ***p<0.001, ****p<0.0001 (one-way ANOVA with Tukey’s post-hoc test).
